# Impact of an Anticaries Mouthrinse on *In Vitro* Remineralization and Microbial Control

**DOI:** 10.1155/2014/982071

**Published:** 2014-02-06

**Authors:** Frank C. Sun, E. Eric Engelman, James A. McGuire, Gabrielle Kosmoski, Lauren Carratello, Danette Ricci-Nittel, Jane Z. Zhang, Bruce R. Schemehorn, Robert J. Gambogi

**Affiliations:** ^1^Johnson & Johnson Consumer & Personal Product Worldwide, Skillman, NJ, USA; ^2^Dental Product Testing, Therametric Technologies, Inc., 9880 Douglas Floyd Parkway, Noblesville, IN, USA

## Abstract

*Objective*. The objective of this research was to evaluate the caries control potential of a new fluoride mouthrinse that also contained antimicrobial agents and a biofilm disrupting agent using different *in vitro* models. *Methods*. Four *in vitro* studies were conducted to assess the performance of this three pronged approach to caries control: (1) traditional enamel fluoride uptake, (2) surface microhardness study using pH cycling model and subsequent fluoride uptake, (3) a salivary biofilm flow-through study to determine the anti-microbial activity, and (4) a single species biofilm model measuring effect on biofilm matrix disruption. *Results*. The data showed that a LISTERINE rinse with fluoride, essential oils and xylitol was superior in promoting enamel fluoride uptake and in enhancing antimicrobial activity over traditional commercially available fluoridated products. An increase of the surface microhardness was observed when the LISTERINE rinse was used in combination with fluoridated toothpaste versus the fluoridated toothpaste alone. Finally, it was demonstrated that xylitol solutions disrupted and reduced the biovolume of biofilm matrix of mature *Streptococcus mutans*. *Conclusion*. These *in vitro* studies demonstrated that a fluoride mouthrinse with antimicrobial agent and biofilm matrix disrupting agent provided multifaceted and enhanced anti-caries efficacy by promoting remineralization, reducing acidogenic bacteria and disrupting biofilm matrix.

## 1. Introduction 

Fluoride has long been used for the prevention and treatment of dental caries, and widespread use of fluoride is a key component to the decline of dental caries around the world [1]. The decline of dental caries can be considered as a major public health achievement, but the burden of the disease is still considerable within all age groups [2]. In the US Department of Health and Human Services' publication “National Call to Action to Promote Oral Health,” dental caries is the single most common chronic childhood disease [3]. Building upon the momentum created by the US Surgeon General's report, the first International Conference on Novel Anticaries and Remineralizing Agents (ICNARA 2008) was held to explore underutilized novel anticaries and remineralization agents for caries prevention and treatment [4]. The technologies that were highlighted included casein phosphopeptide-amorphous calcium phosphate, calcium sodium phosphosilicate, xylitol, antimicrobial peptides, and probiotics [5–9].

Built to the success of the first conference, a second ICNARA conference (2012), was organized to identify the current consensus of thought and remaining questions on pivotal issues in the field of caries prevention [10]. An output of these proceedings highlights that comprehensive caries-prevention protocols should encompass not only agents that affect the remineralization and demineralization process, but also antimicrobial strategies [11]. In addition to the combination of these two strategies, it is proposed to include a third method to disrupt the biofilm matrix of oral bacteria by the use of nonfermentable sugar alcohols [12, 13]. While each treatment method has its own mechanism of action, further evaluation may be required to demonstrate the benefits of combining different approaches for the treatment and prevention of caries [14]. The benefits of fluoride are multifaceted. Once delivered, fluoride can remain in the plaque and saliva and can promote remineralization process [15, 16], incorporate into the demineralized enamel to improve future acid resistance [17, 18], and inhibit demineralization by reducing the activity of the cariogenic bacteria [19]. The potential benefit of using fluoride in combination with antimicrobial agents such as chlorhexidine, triclosan, or essential oils is the reduction of cariogenic microorganisms and consequently plaque acidogenicity [20]. These microorganisms are responsible for the generation of the acids which lead to the demineralization and weakening of the enamel [21]. To further prevent microorganisms from producing acid, xylitol, a non-fermentable sugar alcohol, was incorporated as it has been shown to reduce the adhesion of *S. mutans*, thus giving it the ability to disrupt the biofilm structure [22].

To demonstrate the benefit of this approach, four different *in vitro* studies were conducted: (1) enamel fluoride uptake, (2) a salivary biofilm flow-through study to determine the antimicrobial activity, (3) surface microhardness study using pH cycling model, and (4) a single species biofilm assay measuring the disrupting effect on biofilm matrix.

## 2. Materials and Methods

### 2.1. Test Materials

Tables 1 and 2 contain the available formulation details for the different mouthrinses and toothpastes used in the enamel fluoride uptake (EFU), surface microhardness (SMH) and mixed species flow-through studies. Xylitol solution and sterile water were used in the biofilm disruption assay.

### 2.2. Enamel Fluoride Uptake (EFU) [23]

This test protocol is similar to the one identified as Procedure 40 in the US Food and Drug Administration's 2003 Monograph on anticaries drug products for over-the-counter human use. The modification from the published procedure involves the formation of the incipient caries lesion.

#### 2.2.1. Specimen Preparation

Twelve specimens were evaluated per treatment group. Enamel specimens were cut from human permanent molar teeth that were visually inspected for cracks, exposed dentin, and lesions at 10x magnification to ensure that the substrates do not contain any imperfections prior to preparation. Circular cores were prepared by cutting perpendicularly to the labial surface with a hollow-core diamond drill bit. The resulting circular cores were 3 mm in diameter with at least 0.5 mm of enamel thickness. The cores were mounted onto acrylic rods using methyl methacrylate cold mounting resin. The enamel specimens were polished using 600 grit silicon carbide (SiC) wet/dry sandpaper followed by final polishing using 1 *μ*m gamma alumina compound (LECO, St. Joseph, MI, USA). The polished specimens were visually inspected again for possible imperfections. The cutting and polishing were performed under constant water cooling to prevent overheating of the specimens.

#### 2.2.2. Pretreatment of Specimens

The prepared enamel substrates were etched by immersion into 0.5 mL of 1 M perchloric acid (HClO_4_) for 15 seconds. Throughout the etching period, the etching solutions were continuously agitated. A sample of the etching solution was taken and buffered with total ionic strength adjustment buffer (TISAB) to a pH of 5.2 (0.25 mL sample, 0.5 mL TISAB, and 0.25 mL 1 N sodium hydroxide (NaOH)) and the fluoride content was determined by comparison to a similarly prepared standard curve (1 mL standard + 1 mL TISAB). To calculate the amount of enamel removed by the etching procedure, the calcium content of the etching solution was determined by taking 50 *μ*L of the solutions and analyzing them for calcium by atomic absorption (50 *μ*L q.s. to 5 mL). The atomic absorption results allowed for the indigenous fluoride level of each specimen to be calculated prior to treatment.

Specimens were ground and polished again as described above. An incipient lesion was formed in each enamel test substrate by immersion into a solution that was made up of 0.1 M lactic acid and 0.2% Carbopol 907 (Lubrizol Advanced Materials, Inc., Cleveland, OH, USA) solution and was 50% saturated with hydroxyapatite (HAP) at a pH of 5.0 for 24 hours at 37°C. Finally, the specimens were rinsed with deionized water and stored in a humid environment till needed.

#### 2.2.3. Test Procedure

Groups were created by evenly distributing specimens based on their indigenous fluoride level. Fluoride levels in the enamel were measured before and after treatment. Treatment of the substrates was performed using test products at full concentration without dilutions. Twelve specimens were immersed into 25 mL of test solution with constant stirring (350 rpm) for 30 minutes. The amount of sample was selected as it was allowed for adequate coverage of the substrates. At the end of the treatment period, the specimens were rinsed with deionized water. One layer of enamel was removed from each specimen and analyzed for fluoride and calcium using the acid etching method described above.

### 2.3. Surface Microhardness (SMH) and Fluoride Uptake Post pH Cycling

#### 2.3.1. Specimen Preparation

Eighteen specimens were evaluated per treatment group. The sample size of 18 specimens per treatment provided greater than 90% power for any effect size (difference in treatment means divided by standard deviation) of 1.3 or higher, based on a two-sided *t*-test at the 0.05 level of significance. In addition to being prepared in the same manner listed above in Sections 2.2.1 and 2.2.2, to qualify for testing, enamel substrates must have a measured surface microhardness between 320 and 400 Vickers hardness numbers (VHNs) to ensure that the enamel was sound. Once qualified, an incipient lesion was formed in each enamel test substrate by immersing into a solution that was made up of 0.1 M lactic acid and 0.2% Carbopol 907 solution and was 50% saturated with hydroxyapatite at a pH of 5.0 for 68–96 hours at 37°C.

Baseline SMH for the lesioned specimens, was evaluated with four indentations using a Vickers hardness indenter (at a load of 200 g for 15 seconds). An average of the four indentations was calculated per sample. Each sample had to meet the following criteria: (1) SMH range: 25–45 VHN, (2) standard deviation per chip: ±8.0 VHN, (3) standard deviation per group of 18 enamel chips: ±5.5 VHN, and (4) all indents made at least 0.5 mm away from the edge of the enamel specimen.

#### 2.3.2. Test Procedure

**Figure 1:**
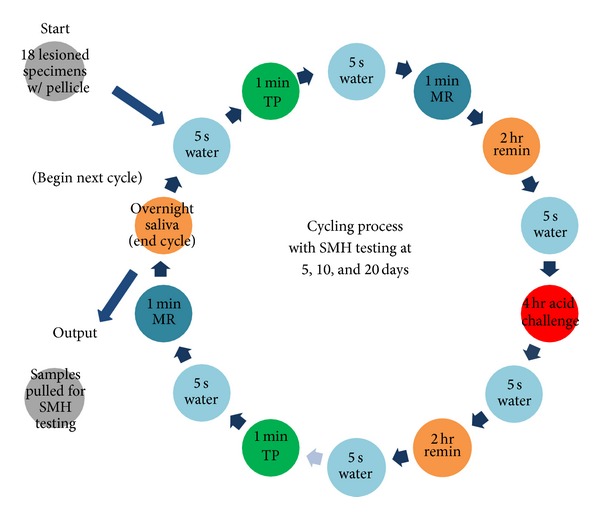
Daily treatment regimen for enamel substrates in the SMH study. TP: toothpaste. MR: mouthrinse.

Groups were balanced based on baseline SMH values. Eighteen enamel specimens from each treatment group were mounted onto specimen holders for treatment. Samples were exposed to saliva for one hour to allow for the formation of pellicle before the first product treatment. The substrates were submitted to a daily treatment regimen consisting of exposure to toothpaste slurry for one minute and rinsing in water for 5 secs, immediately followed by mouthrinse treatment for one minute. This regimen was performed twice per day with one 4-hour acid challenge. Remineralization in human saliva occurred in between treatments. The daily treatment regimen is illustrated in Figure 1.

The toothpaste slurry was created by mixing 5.0 g of the dentifrice with 10 mL of deionized water. Mouthrinses were used undiluted and normalized to 15 mL aliquots for all products, regardless their use directions. Each aliquot was transferred just prior to each treatment. During immersion in both treatment types, the slurry and solution were stirred at 350 rpm. The regimen was repeated for 20 days, with surface microhardness measured at baseline, 5, 10, and 20 days.

EFU was measured after 20 days of pH cycling. The fluoride content was determined using the microdrill technique to a depth of 100 *μ*m. Enamel Fluoride Uptake was calculated as *μ*g F/cm^3^: (*μ*g F × dilution factor/volume of drilling). The ability of the fluoride dentifrice plus test mouthrinse system to promote fluoride uptake was compared to that of the negative and positive controls. After the EFU measurement, all 18 specimens underwent two hours of Simulated Plaque Acid Challenge (SPAC) and surface microhardness was analyzed again to determine the resistance to acid challenge. The SPAC solution was the same acid solution used to form the baseline lesion.

### 2.4. Mixed Species Flow-Through Biofilm Assay for Antimicrobial Activity

#### 2.4.1. Sample Preparation

Eight mouthrinse samples and water were tested against a human salivary mixed species biofilm grown under flow conditions [24]. Oral biofilms were grown on polystyrene pegs (Nunc-TSP Catalog number 445497) which fit into a custom- made 12- channel cassette with stainless steel inlet and outlet ports. Parafilm-stimulated saliva was collected and pooled from 12 healthy donors, who had refrained from oral hygiene for 12 hours. The polystyrene peg lid was placed in the pooled saliva at 30.5°C for 30 minutes to allow for pellicle formation. After pellicle formation, a mixture of remaining saliva and Basic Media was circulated through the cassette for 24-hours to allow for initial biofilm formation on the peg lid.

#### 2.4.2. Test Procedure

Following the initial 24 hour incubation period, the biofilm-coated peg lid was removed from the cassette and immersed in a 96-well microliter plate containing test treatments. Treatments were performed twice daily, based on usage instructions of the product. Five treatments were performed over the course of 60 hours. Biofilm-coated peg lids were reimmersed into a fresh saliva media mixture following each treatment course. Immediately after the final treatment, biofilms were rinsed and harvested via sonication (Sonicator Ultrasonic Processor XL, MISONIX Inc.). Aliquots were washed with 0.1% peptone water and analyzed for ATP (Berthold luminometer (Bad Wildbad, Germany) and Celsis rapid screen ATP bioluminescence reagents (Catalog number 1230941)). Results were reported as log values of relative light units (RLU) per well. The RLU value represented the amount of viable cells.

### 2.5. *Streptococcus mutans* Biofilm Growth for Biofilm Matrix Disruption Assay Using Confocal Laser Scanning Microscopy

#### 2.5.1. Sample Preparation

A *Streptococcus mutans* UA159 planktonic culture was grown aerobically for 24 hours in Brain Heart Infusion (BHI) broth at 33°C. A 3 mL aliquot of sterilely prepared saliva (complete saliva, Northeast Laboratories, Waterville, ME, USA) was added to 35 mm Petri plates (35 mm × 10 mm Sterile Polystyrene Cell Culture Dish, Corning Incorporation, Corning, NY, USA) and incubated aerobically at 33°C for 30 minutes to allow for pellicle formation. The saliva was removed from the Petri plates, and 3 mL of Jordan's media enriched with 3.5% sucrose, 30 *μ*L of Alexa Fluor 647 Dextran Conjugate stain (Invitrogen, Life Technologies Incorporation, Eugene, OR, USA), and 150 *μ*L of the 24-hour culture of *S. mutans* UA159 were dispensed to each Petri plate for each test treatment. The plates were incubated at 33°C aerobically for 24 hours.

#### 2.5.2. Test Procedure

Following biofilm formation, one 60-second treatment was applied to the biofilms while shaking (IKA Microtiter Plate Shaker, Wilmington, NC, USA) at 500 rpm. Treatments were removed by transfer pipette and stain was added for 30 min. Syto 9 green fluorescence nucleic acid stain [25] (Invitrogen, Life Technologies Incorporation, Eugene, OR, USA) was applied to stain bacterial cells. Alexa Fluor 647 Dextran Conjugate was applied during sample preparation to allow time for the stain to be incorporated into the exopolysaccharide (EPS) matrix over the course of biofilm development. The structural organization of the treated biofilms was examined by confocal scanning laser microscopy (CSLM) using a Leica TCS SP5 (Leica Microsystems, Germany) upright microscope with an HCX APO L 63x/0.90 W water immersion lens. The Syto 9 stain was excited by an argon laser at 488 nm wavelength and the Alexa Fluor 647 Dextran Conjugate stain was excited by a helium-neon laser at 633 nm. Five *z*-stacks (*XYZ* orientation: horizontal slices at 512 × 512 pixels) were taken for each biofilm on each Petri plate as representative images. The *z*-step size was 1 *μ*m each. Images were analyzed by Volocity 3D Image Analysis Software (version 6.1, Perkin Elmer, Waltham, MA, USA) for biovolume. Each sample had an *n* = 5.

### 2.6. Statistical Analysis

#### 2.6.1. Enamel Fluoride Uptake

Treatments fluor were compared using analysis of covariance (ANCOVA) model with treatment as the factor and the pretreatment fluoride level as the covariate. Pairwise comparisons for each mouthrinse versus water (negative control) were made at the 0.05 significance level, two-sided. Pairwise comparisons for LISTERINE Advanced Defence Cavity Guard versus each of the other anticavity rinses were made in a sequential manner, ensuring that the familywise error rate was controlled at 0.05.

#### 2.6.2. Surface Microhardness (SMH)

ANCOVA model was used with the change of surface microhardness from baseline to 20 days as the factor and the enamel fluoride uptake from baseline to 20 days as the covariate. Comparisons were made at the 0.05 level, two-sided.

#### 2.6.3. Mixed Species Flow-Through Biofilm Assay for Antimicrobial Activity

The average log RLU values were calculated and ANCOVA model was used with the treatments as the factor and the water negative control as the covariate. Comparisons were made at the 0.05 level, two-sided.

#### 2.6.4. *Streptococcus mutans* Biofilm Growth for Biofilm Matrix Disruption Assay Using Confocal Laser Scanning Microscopy

All *z*-stacks from each test treatment were averaged and reported graphically. Statistical analysis was not conducted on this data.

## 3. Results

### 3.1. Enamel Fluoride Uptake

**Figure 2:**
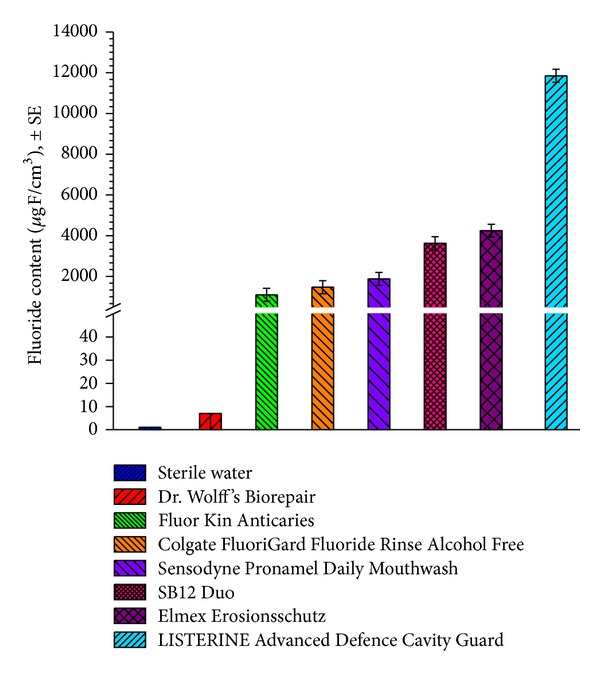
Results of the mean enamel fluoride uptake study (*n* = 12 specimens).

The objective of the study was to determine the effect of the mouthrinses on promoting fluoride uptake into incipient enamel lesions. LISTERINE Advanced Defence Cavity Guard was compared with six commercially available mouthrinses and a negative control (sterilized deionized water). The six commercially available mouthrinses were SB12, Fluor Kin Anticaries, Colgate FluoriGard Fluoride Rinse Alcohol Free, Sensodyne ProNamel Daily Mouthwash, Dr. Wolff's Biorepair, and Elmex Erosionsschutz (Figure 2).

LISTERINE Advanced Defence Cavity Guard (*P* < 0.001), SB12 (*P* < 0.001), Fluor Kin Anticaries (*P* = 0.016), Colgate FluoriGard Fluoride Rinse Alcohol Free (*P* = 0.001), Sensodyne ProNamel Daily Mouthwash (*P* < 0.001), and Elmex Erosionsschutz (*P* < 0.001) resulted in significantly higher levels of fluoride uptake than the negative water control. Dr. Wolff's Biorepair (*P* = 0.988) was found to be equivalent to the negative control in promoting fluoride uptake into enamel lesions.

In comparison to the other mouthrinses tested, LISTERINE Advanced Defence Cavity Guard demonstrated superiority (*P* < 0.001) to each rinse evaluated in promoting fluoride uptake into enamel using the modified version of US FDA test Procedure 40 described in the anticaries monograph.

### 3.2. Remineralization/Demineralization and Enamel Fluoride Uptake Post 20 Days pH Cycling

**Figure 3:**
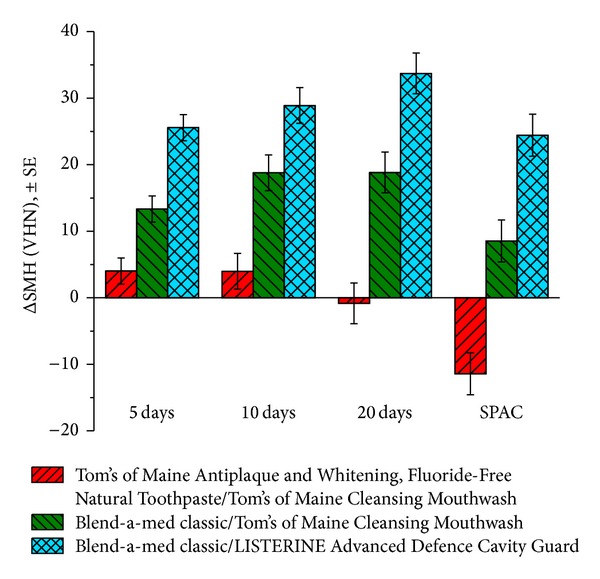
Change in surface microhardness (after baseline) (*n* = 18 specimens per group).

The purpose of this surface microhardness study was to determine the remineralization efficacy and enamel fluoride uptake by using a fluoride mouthrinse in combination with a fluoride toothpaste compared to a fluoride toothpaste plus fluoride-free mouthrinse regimen using a pH cycling protocol. The study included three different treatment regimens: (1) fluoride-free toothpaste/fluoride-free mouthrinse (negative control), (2) fluoride toothpaste/fluoride-free mouthrinse (positive control), and (3) fluoride toothpaste/fluoride mouthrinse. The products used in the study were Blend-a-med classic (fluoride toothpaste), Tom's of Maine Antiplaque and Whitening, Fluoride-Free Natural Toothpaste (fluoride-free toothpaste), LISTERINE Advanced Defence Cavity Guard (fluoride containing mouthrinse), and Tom's of Maine Cleansing Mouthwash (fluoride-free mouthrinse) (Figure 3).

LISTERINE Advanced Defence Cavity Guard showed a statistically significant difference (*P* < 0.001) in ΔSMH at 5, 10, and 20 days compared to the positive and negative controls.

**Figure 4:**
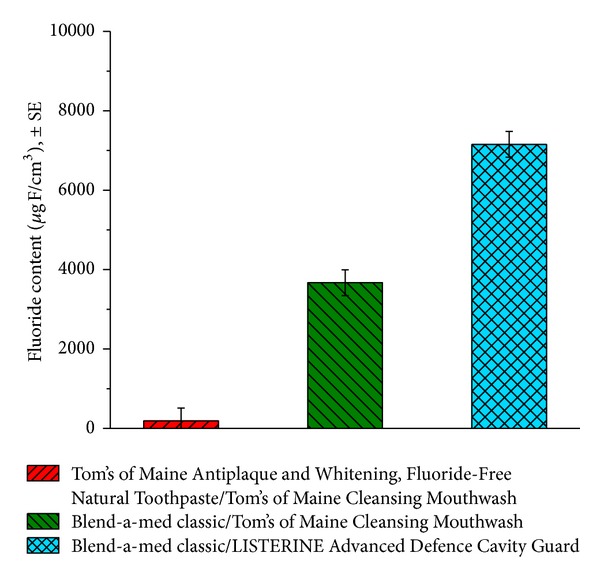
Mean fluoride content (*n* = 18 specimens) after 20 days of the pH cycling protocol.

Enamel Fluoride was analyzed using the microdrill after 20 days of pH cycling to further demonstrate the benefit of the combination of a fluoride toothpaste and fluoride mouthrinse (Figure 4).

LISTERINE Advanced Defence Cavity Guard showed a statistically significant difference (*P* < 0.001) 20 days after fluoride measurement compared to the positive and negative controls. This single point of data along with the SMH results indicated that there was additional benefit of using fluoride mouthrinse in combination with fluoride toothpaste versus fluoride toothpaste alone.

### 3.3. Mixed Species Flow-Through Biofilm Assay for Antimicrobial Activity

The mixed species flow-through study was performed to demonstrate the antimicrobial activity of LISTERINE Advanced Defence Cavity Guard in comparison to seven commercially available mouthrinses and a negative control (filter-sterilized deionized water). The commercially available mouthrinses were SB12 Duo, Fluor Kin Anticaries, Colgate FluoriGard Fluoride Rinse Alcohol Free, Sensodyne ProNamel Daily Mouthwash, Dr. Wolff's Biorepair, Elmex Erosionsschutz, and Flux Mot Karies.

**Figure 5:**
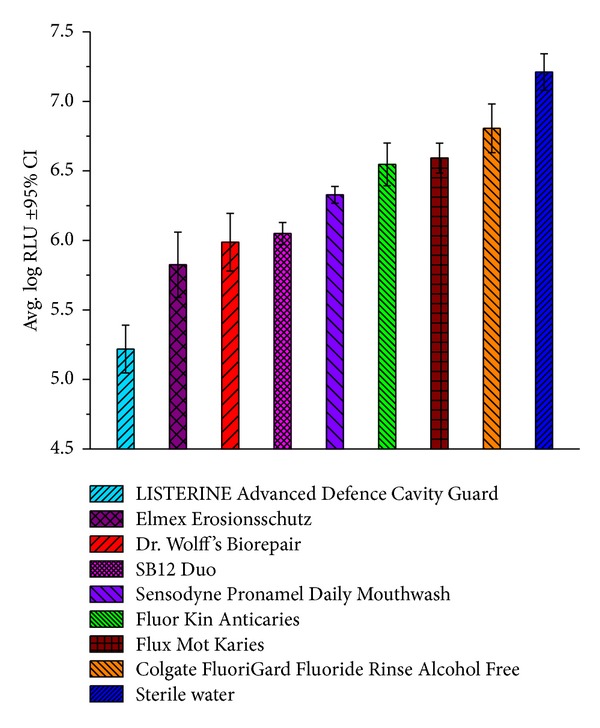
Mixed species flow-through biofilm assay for antimicrobial activity.

LISTERINE Advanced Defence Cavity Guard had an average log RLU of 5.22. All other commercially available fluoridated mouthrinses ranged from an average log RLU of 5.82 to 6.81 (Figure 5). Statistically, each rinse had significantly lower log RLU than water (*P* = 0.0017) for FluoriGard versus water and *P* < 0.001 for all other rinses versus water. In comparison to the other mouthrinses, LISTERINE Advanced Defence Cavity Guard had a significantly lower log RLU than each competitor rinse (*P* < 0.001). A lower log RLU represents a lower amount of viable bacteria.

### 3.4. *Streptococcus mutans* Biofilm Growth for Biofilm Matrix Disruption Assay Using Confocal Laser Scanning Microscopy

**Figure 6:**
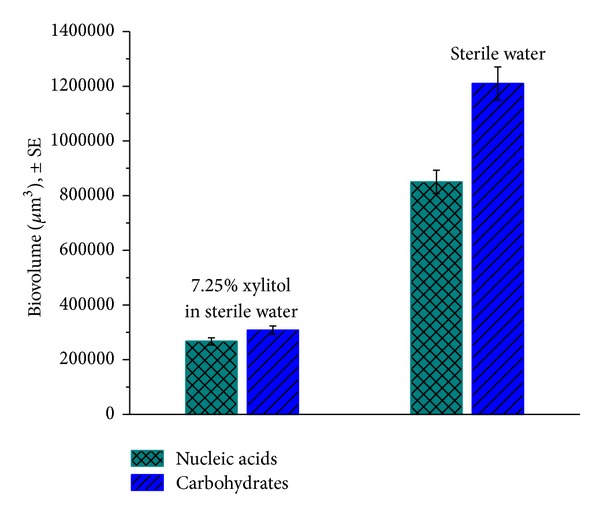
Biovolume of *S. mutans* UA159 biofilm components following a single 60-second exposure of treatment with 7.25% xylitol solution or sterile water.

Biovolume (*μ*m^3^) for both the Syto 9 and Alexa Fluor 647 Dextran Conjugate stain was quantified. All fluorescent data collected from the five *z*-stacks were averaged. The results shown in Figure 6 are of the nucleic acids and dextran polysaccharides after a single 60-second exposure of either a simple solution of 7.25% xylitol in sterile water or sterile water treated biofilms.

A single 60-second treatment of a 7.25% aqueous xylitol solution on a 24-hour *S. mutans* biofilm showed a greater reduction in overall carbohydrates compared to sterile water treated biofilm (biovolume of 3.08 × 10^5^ *μ*m^3^ and 1.21 × 10^6^ *μ*m^3^, resp.). Nucleic acids also showed a reduction (biovolume of 2.67 × 10^5^ *μ*m^3^ and 8.50 × 10^5^ *μ*m^3^, resp.).

**Figure 7:**
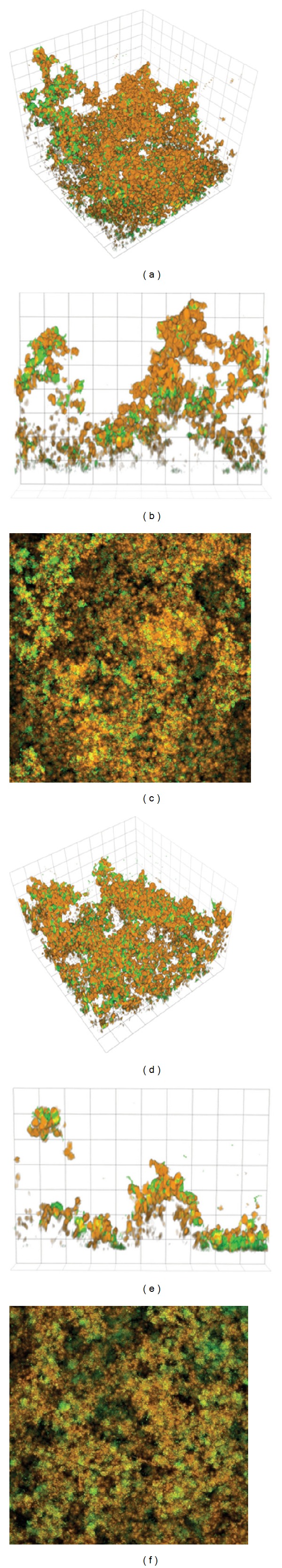
Representative images of sterile water treated *S. mutans* biofilms ((a), (b), (c)) and xylitol solution treated *S. mutans* biofilms ((d), (e), (f)). Alexa Fluor 647 Dextran Conjugate stain (carbohydrates) is represented in orange. Syto 9 stain (nucleic acids) is represented in green. Figures (a) and (d) depicted 3D tilted side views and (b) and (e) show 3D side slice view (10 *μ*m). Figures (c) and (f) showed top to botton views of 2D extended focus views.

Visual assessment of the treated biofilms indicated that xylitol solution treated biofilms were thinner and sparser (Figure 7(d)) and showed less EPS overall than the sterile water treated biofilms. Sterile water treated biofilms show a fuller, taller, and denser biofilm (Figure 7(a)). These assessments were visualized in both the 3D-tilted side view and the 3D 10 *μ*m slice side view, as seen in Figures 7(a)–7(f).

## 4. Discussion

The anticaries benefits of fluoride have been proven extensively for a variety of oral care treatments from toothpastes, mouthrinses, and varnishes, to gels [26]. However, the systematic review of the use of sodium fluoride mouthrinses in controlled clinical trials by Twetman et al. concluded that there was limited evidence that daily or weekly rinsing with a fluoride mouthrinse had a significant caries-reducing effect on young permanent teeth compared with placebo [27]. In another review, Marinho et al. evaluated five different trials involving fluoride toothpaste plus fluoride mouthrinse versus toothpaste alone (*n* = 2738) [1]. While not in contrast with Twetman et al.'s conclusions, the results of Marinho's random-effects-meta-analysis of the five trials found that the trials have a combined prevented fraction pooled estimate of 0.07 (95% CI, 0.00 to 0.13; *P* = 0.06). This result was directionally in favor of the combined regimen (fluoride toothpaste plus fluoride mouthrinse) within a relatively narrow confidence interval for pooled estimate of effect. One would predict that an upgrade in mouthrinse formulation would further enhance treatment effect.

While true clinical significance has yet to be shown for the use of a fluoride mouthrinse, studies have reported an inverse relationship between salivary fluoride concentration and dental caries prevalence and severity in primary teeth [28, 29]. A study by Duckworth et al. used salivary fluoride clearance measurements to determine the consequences of flossing and mouth rinsing on the delivery of fluoride from a fluoride toothpaste [30]. The conclusion of Duckworth's study was that the combination of fluoridated toothpaste and fluoridated mouthrinse may be beneficial against caries, as it delivered significantly more fluoride to saliva when compared to toothpaste alone or toothpaste followed by professional flossing. Other studies found that the frequency of the use of the fluoride mouthrinse and the regular elevation of fluoride in the oral fluids to maintain an optimal fluoride concentration were important for caries control [31, 32].

Perhaps an important factor to consider is the delivery and the biological availability of fluoride from the oral care treatments. It is acknowledged that excipients in a toothpaste formulation could reduce the anticaries performance [33]. Similar interactions could affect the anticaries performance of mouthrinse. Faller et al. found that fluoride containing mouthrinses formulated with the same level of sodium fluoride did not perform equally in their *in vitro* testing [34]. Faller concluded that the bioavailability of fluoride in mouthrinses was influenced by formulation pH, as well as sodium lauryl sulfate (SLS). While it is clear that some ingredients may have negative effects on fluoride bioavailability, a study by Moi et al. demonstrated that other ingredients may have a neutral effect [35]. Their findings showed that their CPC containing fluoride mouthrinse had similar anticaries potential as that of the positive control, hence concluding that their formulation components did not reduce bioavailability of the fluoride.

To develop a treatment with a greater anticaries potential, agents that affect the dental hard tissue and the microbial biofilms must be combined together. Based on this combination approach, fluoride has been combined with essential oils and xylitol for a treatment regimen that affects remineralization and demineralization, controls cariogenic bacteria, and disrupts biofilm matrix. LISTERINE Advanced Defence Cavity Guard (LISTERINE Advanced Defence Cavity Guard is also known as LISTERINE Professional Fluoride Plus or LISTERINE Professional Cavity Guard) was designed to utilize this three-pronged strategy for caries prevention.

The *in vitro* EFU study was conducted to determine the effect of different commercially available mouthrinses on promoting fluoride uptake into incipient enamel lesions. The results of the EFU study showed that the LISTERINE Advanced Defence Cavity Guard demonstrated superiority to each commercially available rinse evaluated in this assay. The lower pH of the formulation and the absence of components that interfered with fluoride bioavailability were the key factors for the improved EFU results for LISTERINE Advanced Defence Cavity Guard. Though the fluoride level in LISTERINE Advanced Defence Cavity Guard was lower, similar or higher than other tested mouthrinses, as shown in Table 3, it consistently provided higher enamel fluoride uptake in this *in vitro* study. In other words, more fluoride was retained on the enamel surface because of the way LISTERINE Advanced Defence Cavity Guard was formulated. It has been noted by Vogel et al. [36] that fluoride reservoirs can provide a benefit in preventing caries. Therefore, we believe this elevated EFU level could help provide a cariostatic effect to the teeth.

The anticaries efficacy of fluoride containing toothpaste and mouthrinse individually has been well established [37]. However, there are inconsistent results of the benefit of fluoride mouthrinse in addition to fluoride toothpaste [38]. The *in vitro* cycling study presented here was designed to assess the incremental remineralization of the artificial lesion and enamel fluoride uptake of fluoride mouthrinses along with fluoride toothpaste versus fluoride toothpaste alone. This was done in comparison to a fluoride toothpaste/fluoride-free mouthrinse after pH cycling. This model was chosen as it has been shown to correlate with clinical efficacy measurements [39].

A pH cycling model simulates the dynamic variations in mineral saturation and pH associated with the natural caries process [40]. The model has been validated through fluoride dose response and surface microhardness [41, 42]. The model allows for controlled experimental conditions, dental substrate selection, and acid challenge solution that simulates plaque [43]. This model also allows for testing the preventive efficacy or treatment efficacy of multiple formulations or regimens in the same study.

Though they are widely used for mechanistic research or product profiling, pH cycling models often have varied experimental conditions, that is, daily treatment, the length of each treatment, the days of cycling, the baseline microhardness, the number of specimens in each treatment group, and so forth. The experimental parameters are typically varied to address the scientific question of interest. The experimental conditions utilized in this study are valid, based on the fact that both 20-day mean microhardness and fluoride uptake were statistically significantly higher for fluoride toothpaste plus fluoride-free mouthrinse than for the negative control. It should be noted that after the SPAC treatment, there was a significant reduction of the microhardness for fluoride-free treatment group, indicating the vulnerability of the enamel surface with the absence of fluoride in any treatment modality.

The study was considered valid, as both the 20-day microhardness mean change and mean fluoride uptake were statistically significantly higher for the positive control (fluoride toothpaste plus fluoride-free mouthrinse) than for the negative control (fluoride-free toothpaste plus fluoride-free mouthrinse).

While the SMH data and the EFU in remineralization/demineralization model have already demonstrated a significant remineralization effect with addition of LISTERINE mouthrinse over toothpaste alone, the true benefit of this three-pronged treatment technique may not be discernible in this *in vitro* model. The challenge stems from the use of a chemical-based demineralization solution in the current study. The anti-caries potential of products that have an anti-microbial component and a biofilm matrix disrupter would be fully reflected by biological remineralization/demineralization cycling.

Mixed species flow-through biofilm assay for antimicrobial activity was capable of discriminating differences among treatments with differing levels of antiseptic activity, as well as differing active ingredients based on differing mechanisms of action [44]. The results showed that the essential oil and xylitol containing mouthrinse was the most effective formulation against mixed species saliva-derived biofilms, independent of type of treatment or age of biofilm.

Many clinical studies have been conducted to evaluate the anticariogenic effect of xylitol [45]. Xylitol is well known to have the ability to reduce the levels of *Streptococcus mutans* due to its inability to properly metabolize xylitol [46]. This inability to metabolize xylitol prevents the production of lactic acid, preventing this pathway of demineralization. However, this anticaries method requires the constant delivery of xylitol to maintain a therapeutic concentration in the mouth. The challenge was to determine the optimum dose per day to deliver an anticaries benefit and evaluate possible delivery vehicle options [47]. It should be noted that the short-term exposures of mouthrinse to biofilm in the studies discussed here were not designed to explicitly evaluate the impact of xylitol on the fermentation process. However, the impact of xylitol on biofilm disruption was probed as shown in Figure 7.

After one 60-second treatment in the biofilm matrix disruption assay, the decrease in biovolume measured showed the ability of xylitol to disrupt the biofilm matrix. The xylitol containing solution was able to reduce the dextran carbohydrates by an order of magnitude, which could be seen in Figure 7. The reduced amount of biofilm was hypothesized to be proportional to a reduction of bacterial acid production, which could lead to a reduction of demineralization. A future direction for this study would be to introduce a challenge of sucrose and xylitol, allow a period of time for the micro-organisms to generate matrix and plaque acids, and treat the samples with fluorinated mouthrinse and measure the SMH or EFU values of the surface. This may allow for improved quantification of the anti-caries potential from disrupting the biofilm matrix. This finding was significant because it was believed that reducing the amount of biofilm matrix could be of benefit in controlling cariogenic biofilms.

The assumptions of the models discussed here are that they will help provide direction toward delivering improved clinical outcomes. However, not all *in vitro* study outcomes discussed here have been validated with respect to human clinical studies. The studies above are the initial evidence to encourage further exploration into the philosophy of multiple mechanisms acting together for caries prevention. The authors realize that additional studies are warranted to continue delving into the benefits of using multiple mechanisms in combination. Some examples of future studies that are being contemplated are (1) developing a cariogenic biofilm to be employed as the demineralization agent to allow one to see the benefit of having a matrix disrupter and/or antimicrobial agent to enamel structure, (2) Assessment of combination technologies in an *in-situ *assay and finally (3) developing validated relationships of *in vitro* study results with clinically relevant outcomes.

## 5. Conclusions

LISTERINE Advanced Defence Cavity Guard demonstrated superiority to each comparative rinse evaluated in promoting fluoride uptake. This new LISTERINE mouthrinse was shown to have an additive effect on remineralization of enamel lesions in a 20-day pH cycling study when used in combination with fluoridated toothpaste. The superior antimicrobial activity of this new LISTERINE mouthrinse was demonstrated over seven other commercially available fluoridated mouthrinse products in a flow through biofilm model. The benefit of inclusion of xylitol in a mouthrinse was demonstrated in confocal microscopy images and the reduction of biovolume.

## Figures and Tables

**Table 1:** Mouthrinses used in the different studies.

Mouthrinse	Ingredients	Study
LISTERINE Advanced Defence Cavity Guard	Aqua, sorbitol, xylitol, propylene glycol, poloxamer 407, sodium Lauryl sulfate, aroma, sodium fluoride, eucalyptol, benzoic acid, sodium benzoate, methyl salicylate, thymol, menthol, sodium saccharin, sucralose	EFU, SMH, Flow Through

SB12 Duo (Antula Healthcare)	Aqua, glycerin, sorbitol, alcohol, zinc acetate, chlorhexidine diacetate, sodium fluoride, hydrogenated castor oil, potassium acesulfame, citric acid, aroma	EFU, Flow Through

Fluor Kin Anticaries (Laboratorios Kin SA)	Aqua, sorbitol, glycerin, PEG-40 hydrogenated castor oil, sodium methylparaben, aroma, citric acid, sodium ethylparaben, sodium fluoride, menthol, sodium saccharin, limonene, CI 47005, CI 42051	EFU, Flow Through

Colgate FluoriGard Fluoride Rinse Alcohol Free (Colgate Palmolive (UK) Ltd.)	Aqua, glycerin, propylene glycol, sorbitol, sodium phosphate, poloxamer 407, sodium benzoate, disodium phosphate, aroma cetylpyridinium chloride, sodium fluoride, sodium saccharin, cinnamal, CI 19140, CI 42053	EFU, Flow Through

Sensodyne ProNamel Daily Mouthwash (GlaxoSmithKline Consumer)	Aqua, glycerin, poloxamer 338, PEG-60 hydrogenated castor oil, VP/VA copolymer, potassium nitrate, sodium benzoate, cellulose gum, aroma, sodium fluoride, methylparaben, propylparaben, cetylpyridinium chloride, sodium saccharin, xanthan gum, disodium phosphate, sodium phosphate, CI 42090	EFU, Flow Through

Dr. Wolff's Biorepair (Dr. Kurt Wolff)	Aqua, sorbitol, alcohol denat., glycerin, xylitol, cellulose gum, zinc PCA, zinc hydroxyapatite, aroma, sodium lauryl sulfate, silica, *Ricinus communis* seed oil, ammonium acryloyldimethyltaurate/VP copolymer, sodium myristoyl sarcosinate, sodium methyl cocoyl taurate, sodium saccharin, sodium benzoate, benzyl alcohol, phenoxyethanol, limonene	EFU, Flow Through

Elmex Erosionsschutz (GABA GmbH)	Aminfluorid, natriumfluorid, aqua, glycerin, aminfluorid, glycerin, sodium gluconate, PEG-40 hydrogenated castor oil, olaflur, aroma, stannous chloride, sodium fluoride, cocamidopropyl betaine, sodium saccharin, hydrochloric acid	EFU, Flow Through

Flux Mot Karies	Aqua, glycerin, alcohol denat., xylitol, polysorbate 80, potassium sorbate, citric acid, sodium saccharin, sodium fluoride, CI 42051, *Mentha piperita *	Flow Through

Tom's of Maine Cleansing Mouthwash (Tom's of Maine (UK) Ltd.)	Water, glycerin, propanediol, poloxamer 335, xylitol, spearmint, benzoic acid, menthol	SMH

**Table 2:** Toothpastes used in the SMH study.

Toothpaste	Ingredients	Study
Blend-a-med classic (Procter and Gamble Balkans)	Aqua, aroma, CI 77891, glycerin, hydrated silica, limonene, natriumfluorid, sodium fluoride, sodium lauryl sulfate, sodium saccharin, xanthan gum, zinc lactate	SMH

Tom's of Maine Antiplaque and Whitening, Fluoride-Free Natural Toothpaste (Tom's of Maine (UK) Ltd.)	Calcium carbonate, water, glycerin, sodium bicarbonate, xylitol, sodium lauryl sulfate, natural flavor, carrageenan, propolis extract, *Commiphora myrrha* (myrrh) resin extract	SMH

**Table 3:** Fluoride and pH of mouthrinses assessed in the *in vitro* EFU and SMH studies.

Mouthrinse	Fluoride (ppm)	Measured pH
LISTERINE Advanced Defence Cavity Guard	450	4.2
SB12 Duo	900	5.7
Fluor Kin Anticaries	226	6.5
Colgate FluoriGard Fluoride Rinse Alcohol Free	225	5.9
ProNamel Daily Mouthwash	450	6.4
Dr. Wolff's Biorepair	No fluoride	
Elmex Erosionsschutz	500	4.5
